# Distinguishing Hemodynamics from Function in the Human LGN Using a Temporal Response Model

**DOI:** 10.3390/vision3020027

**Published:** 2019-06-07

**Authors:** Kevin DeSimone, Keith A. Schneider

**Affiliations:** 1 Department of Psychology, New York University, New York, NY 10003, USA; 2 Centre for Vision Research, York University, Toronto, ON M3J 1P3, Canada; 3 Department of Biology, York University, Toronto, ON M3J 1P3, Canada; 4 Department of Brain & Psychological Sciences, University of Delaware, Newark, DE 19716, USA

**Keywords:** LGN, pRF, spatiotemporal, retinotopic, flicker, isoluminance, clustering

## Abstract

We developed a temporal population receptive field model to differentiate the neural and hemodynamic response functions (HRF) in the human lateral geniculate nucleus (LGN). The HRF in the human LGN is dominated by the richly vascularized hilum, a structure that serves as a point of entry for blood vessels entering the LGN and supplying the substrates of central vision. The location of the hilum along the ventral surface of the LGN and the resulting gradient in the amplitude of the HRF across the extent of the LGN have made it difficult to segment the human LGN into its more interesting magnocellular and parvocellular regions that represent two distinct visual processing streams. Here, we show that an intrinsic clustering of the LGN responses to a variety of visual inputs reveals the hilum, and further, that this clustering is dominated by the amplitude of the HRF. We introduced a temporal population receptive field model that includes separate sustained and transient temporal impulse response functions that vary on a much short timescale than the HRF. When we account for the HRF amplitude, we demonstrate that this temporal response model is able to functionally segregate the residual responses according to their temporal properties.

## 1. Introduction

Over the past 15 years, researchers have characterized the retinotopic organization of the human LGN in vivo [1,2,3,4,5]. Along with its small size and deep location in the brain, the hemodynamic properties of the LGN have made it difficult to study using functional magnetic resonance imaging (fMRI) techniques. Of particular interest for study in the LGN is the development of methods for functionally segmenting its magnocellular (M) and parvocellular (P) layers. The LGN is somewhat unique in the visual pathway in that there is a clear separation of structure and function at a spatial scale resolvable by contemporary functional imaging techniques. Therefore, it provides a unique opportunity for developing and testing models of neural function, visual perception, and information flow throughout the brain. For instance, one prevailing theory of dyslexia contends that a malfunction in the M system throughout the brain is responsible for the behavioral deficits observed in dyslexics [6,7]. A key test of this theory would revolve around the functional segmentation of the LGN in vivo.

Recent attempts have been made to segment the LGN into its M and P subdivisions [8,9,10] using fMRI. In these experiments, researchers took advantage of the complementarity of the response properties of M and P neurons to differentially drive the BOLD activity during the presentation of various stimulus features (i.e., contrast, spatial frequency, temporal frequency, chromaticity). Using a general linear model (GLM), voxels were characterized on the basis of their response preference as gleaned from the regression coefficients for various stimulus properties. Voxels were classified as M or P depending on their BOLD response to M-preferred stimulus features (i.e., low contrast, low spatial frequency, high temporal frequency, achromatic) or to P-preferred stimulus features (i.e., high contrast, high spatial frequency, low temporal frequency, chromatic).

However, to properly account for the neural function in the LGN using fMRI, we must first account for potential differences in the hemodynamics throughout the LGN. Oxygenated blood from the internal carotid artery reaches the LGN by way of the anterior choroid and posterior cerebral arteries. These vessels ramify and enter the LGN at the hilum on its ventral surface about halfway along its medial-to-lateral extent. The hilum is richly vascularized, with the tissue of the LGN surrounding the hilum receiving approximately twice the blood supply as the rest of the LGN [11]. The hilar region of the LGN receives projections from the macular retina and contains foveal and parafoveal representations of the visual field along the horizontal meridians, suggesting a functional role for the rich vascularization of the hilum. This neurovascular organization has been observed in the postmortem human brain, as well as in a number of other specimens including non-human primates, mammals, reptiles, and marsupials [11].

The goal of this study was to develop a spatiotemporal population receptive field (pRF) model to capture the functional divergence between the M and P subpopulations of the LGN. By adding a temporal parameter to the spatial pRF model, it is possible to model and account for variations in neural function at timescales much faster than are typically observed in the slow hemodynamic response. Using a stimulus that varied in visuotopic location and flicker frequency over time, we sought to characterize each voxel both in terms of its spatial and temporal response and in doing so functionally segment the LGN. The parameter maps we measured indicate that this simple modification to the spatial pRF model is able to capture functional differences among the M and P layers of the LGN. In follow up experiments, we compared these model-based functional segmentations of the LGN to model-free, data-driven segmentations. We designed stimuli with no spatial component and attempted to differentiate the subpopulations of the LGN on the basis of the pattern of their activity in response to random changes in flicker frequency, spatial frequency, and isoluminance in a full-field visual stimulus. We compared these cluster labels to our spatiotemporal pRF model estimates to ascertain the effectiveness of functionally segmenting the layers of the LGN on the basis of temporal frequency tuning. The results suggest that (1) a spatiotemporal pRF model can capture functional differences across the M and P layers of the LGN, and (2) that such a model needs to include a scaling term in order to account for the difference in the hemodynamics across the extent of the LGN.

## 2. Experimental Procedures

### 2.1. Subjects

Three subjects participated in the study (28–32 years of age; 2 males, 1 female). One subject was left-handed. The subjects were in good health with no history of psychiatric or neurological disorders, had normal or corrected-to-normal vision, gave their informed written consent, and were compensated for their participation. The study was approved by the York University Human Participants Review Committee, Certificate #2014-300. All of the subjects participated in 10 scanning sessions, including one anatomical and nine functional.

### 2.2. Display Hardware

The stimuli were generated on a Mac Pro i7 computer (Apple, Cupertino, CA, USA) with a NVIDIA Quadro 4000 video card, using Matlab software (The Mathworks, Natick, MA, USA) and Psychophysics Toolbox 3 functions [12,13]. Stimuli were presented using a PROPixx DLP LED projector (VPixx Technologies, Saint-Bruno, QC, Canada) located outside the scanner room and projected through a waveguide and onto a translucent screen located at the end of the scanner bore. Subjects viewed the screen at a total viewing distance of 31 cm through a mirror attached to the head coil. The display subtended approximately 26° of visual angle horizontally and 20° vertically. A trigger pulse from the scanner, which was translated into a key press by the response box, was used to synchronize the start of the stimulus presentation to the beginning of the image acquisition.

### 2.3. Visual Stimuli and Procedure

**Figure 1:**
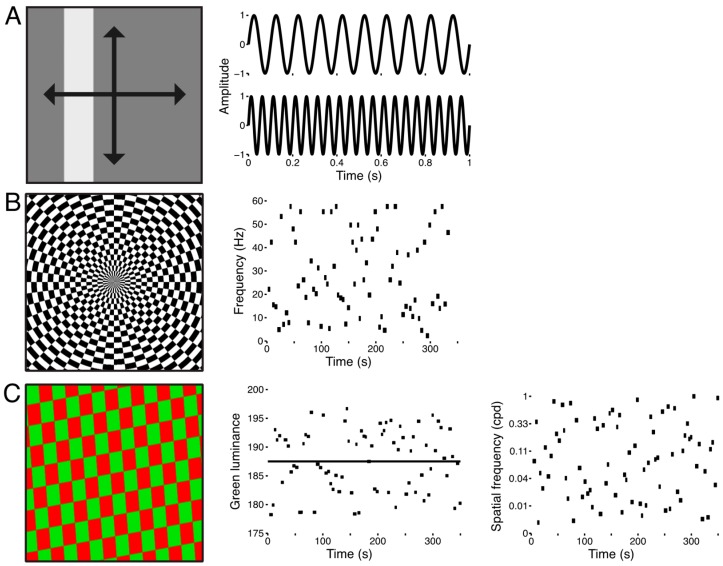
Stimuli used for the three experiments. (**A**) Sweeping bar stimulus. The spatially uniform flickering bar swept across the display 8 times in 4 directions and at 2 flicker frequencies, 10 Hz and 20 Hz. (**B**) Full-field flicker frequency stimulus. The stimulus was a checkerboard that subtended the entire display (left panel). The luminance of each checkerboard element was sinusoidally modulated between black and white. Flicker frequencies were drawn from a uniform distribution from 2–60 Hz and were presented for variable durations of 4–6 s. Each scanning run was comprised of 75 of these trials (right panel). (**C**) Full-field red-green isoluminance stimulus. The stimulus was a full-field checkerboard whose elements were colored red and green (left panel) and which reversed contrast at 15 Hz. We varied the luminance of the green elements (±5%) about the predefined isoluminant reference point (middle panel, black line). We also varied the spatial frequency (right panel) and orientation (not shown) of the checkerboard pattern from trial to trial. Each functional run contained 75 of these trials, and the stimulus was repeated across all scanning runs so that a mean functional run could be computed.

Each subject participated in three separate functional imaging experiments (Figure 1). The stimulus for the first experiment consisted of a uniform bar subtending 5° of visual angle across its width and extending to the display boundary along its length. The bar transited across the visual field in four directions (0°, 90°, 180°, 270°) twice during each scanning run (Figure 1A). The luminance of the bar was sinusoidally modulated between black and white at a rate of 10 Hz for the first four passes and at 20 Hz for the second four passes. The 10 Hz and 20 Hz luminance profiles were sampled and displayed at 480 Hz so as to smoothly sample the sinusoidal luminance modulation. The bar stimulus was presented overtop a uniform mean luminance (i.e., gray) field while subjects fixated a central dot. Each run lasted 5 min.

The stimulus for the second experiment consisted of a checkerboard texture spanning the entire extent of the display (Figure 1B). The luminance of the elements of the checkerboard was sinusoidally modulated between black and white and was sampled at a rate of 1440 Hz. This high temporal precision in the refresh rate of the display allowed for wider selection of flicker frequencies and finer graduation of the sinusoidal luminance modulation. The scanning run was structured into trials of variable durations and flicker rates. Trial durations and flicker frequencies were sampled from uniform distributions spanning 4–6 s and 2–60 Hz. Each run contained 75 of these trials and lasted approximately 5.5 min while subjects fixated a central dot. The same trial structure was used across all scanning runs so that data could be combined across runs and sessions for a given subject.

The stimulus for the third experiment also consisted of a full-field stimulus (Figure 1C), but here we varied the spatial frequency and relative luminance of the elements of a red-green checkerboard texture. The texture subtended the entire display and reversed contrast at 15 Hz. At the outset of each scanning session we used a flicker photometry procedure to determine each subject’s isoluminance for red-green. Subjects were presented with a red-green checkerboard texture that reversed contrast at 15 Hz. Their task was to adjust the green luminance in order to minimize the heterochromatic flicker [14], and we took the mean of 30 such calibration trials to constitute an isoluminant reference point. From trial to trial, we varied the spatial frequency and divergence from isoluminant reference point. Spatial frequencies were uniformly sampled from 0.1–1 cpd in log-space, and the green luminance was varied 5% above and below the empirically determined isoluminant reference point. Each run contained 75 of these trials and lasted approximately 6 min while subjects fixated a central dot.

### 2.4. Data Acquisition

Data were acquired in the York University Neuroimaging Laboratory with a 3 T Siemens Trio MRI scanner (Siemens, Erlangen, Germany) using a 32-channel head coil. Functional series were acquired with 14 coronal slices (1.5 mm isotropic voxels with 0.5 mm gap between slices) and a gradient echo, echo planar sequence with a 128 square matrix leading to an in-plane resolution of 1.5 × 1.5 mm [repetition time (TR), 1.5 s; echo time (TE), 42 ms; flip angle, 90°; foot-to-head phase encoding]. A partial Fourier factor of 7/8 was used to acquire an asymmetric fraction of *k*-space to reduce acquisition time. The posterior edge of the acquisition volume was aligned in the midsagittal plane with the posterior edge of the superior colliculus. The subjects’ heads were surrounded by foam padding to reduce head movements. The distortions commonly found in T2* GE-EPI were significantly attenuated due to the deep location of the structures of interest, the short TE, the small voxels, and the phase-encoding direction.

In addition to the functional scanning sessions, each subject participated in an anatomical scanning session. During the anatomical session, a high-resolution T_1_-weighted MPRAGE (spin-echo, TR = 685 ms, TE = 8.6 ms, flip angle = 75°, 256 square matrix) and 40 coronal proton-density weighted volumes (TR = 3 ms, TE = 26 ms, flip angle = 120°, 19–48 slices, 1–2 mm thick, 256 square matrix, 192 mm field view, 0.375 × 0.375 mm^2^ in-plane resolution, GRAPPA acceleration = 2) were acquired. Each proton-density image was motion-corrected to a single proton-density image, from which a mean proton-density image was computed and up-sampled by a factor of 2. These images were aligned to the MPRAGE and used for drawing ROI.

### 2.5. Population Receptive Field Estimation

The first four volumes of each run were discarded. To compensate for subject head movement, the remaining volumes were registered to a single volume obtained during the same scanning session. In addition to motion-correcting the functional imaging data, we also employed a volume-censoring procedure [15]. The frame-wise displacement is an aggregate measure of the translational and rotational head-movement gleaned from the motion-correction transformation for each volume in the functional series. Volumes with a frame-wise displacement greater than 0.4 mm were flagged for censoring and so were not included in the mean functional series. Data were spatially resampled to twice the native resolution. No spatial or temporal smoothing was applied to the functional data.

**Figure 2:**
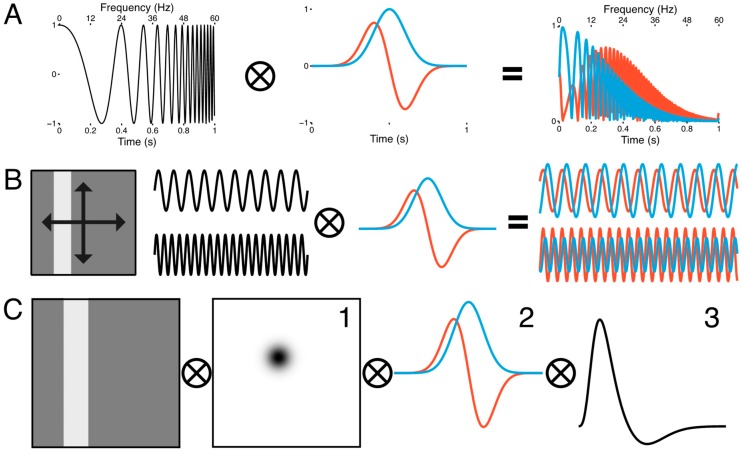
Spatiotemporal population receptive field model. (**A**) The sustained (blue) response is modeled as a Gaussian receptive field in time, while the transient (red) response is modeled as its first derivative (middle panel). When a frequency modulated signal (left panel) is passed through the transient and sustained temporal receptive fields, the amplitude of the output if frequency-dependent (right panel). (**B**) Spatial tuning was encoded with a sweeping, uniform luminance bar stimulus that traversed the visual field twice along four trajectories (left panel). Flicker modulation was embedded in the spatial encoding regime. The luminance of the bar sinusoidally flickered at 10 Hz for the first set of four bar passes and 20 Hz for the second set of four bar passes (middle panels). At 10 Hz, the sustained response had a greater amplitude than the transient response, but at 20 Hz this pattern reversed (right panel). (**C**) The spatial response was extracted by convolving the bar stimulus with a 2D Gaussian (1), which was in turn convolved with the transient and sustained temporal receptive fields (2). This was then convolved with the hemodynamic response function (3) to approximate the BOLD signal.

We modeled the BOLD signal of each voxel in terms of a spatiotemporal population receptive field. The spatial response of each voxel was characterized as a two-dimensional Gaussian in visuotopic coordinates [16,17]. It describes the location and extent of the BOLD response of a given voxel, formulated as

$$R=\mu +\beta \times \int^{\;}S\left(x,y,t\right)*G\left(x,y\right)dxdy$$

$$G\left(x,y\right)=e^{-\frac{{\left(x_{0}-x\right)}^{2}+{\left(y_{0}-y\right)}^{2}}{2\sigma^{2}}}$$

where S is the stimulus and G is the Gaussian with center (x, y) and standard deviation σ. The parameter β is a scaling factor to account for the arbitrary units of the BOLD response. The parameter μ accounts for the global mean of each voxel and serves as a baseline correction. To this spatial pRF model, we add a temporal component to capture the differences in the discharge pattern across the M and P neural subpopulations of the LGN. Among their many complementary stimulus response properties, M and P neurons of the macaque LGN have been shown to functionally diverge on the basis of the temporal profile of their responses [18,19,20], with P neurons exhibiting a sustained response to a light stimulus and M neurons exhibiting a transient response. The temporal component of the pRF model takes advantage of this functional divergence between and M and P neurons and models the BOLD response of a given voxel as a weighted sum of a pair of transient and sustained receptive fields responses to a flickering visual stimulus. The temporal pRF is formulated as

$$R_{S}\left(t\right)=e^{-\frac{{\left(t-t_{0}\right)}^{2}}{\sqrt{2\pi \tau }}}$$

$$R_{T}\left(t\right)={R_{S}}^{\prime }$$

where the sustained response $R_{S}$ is a one-dimensional Gaussian spanning the duration of a single volume acquisition centered at time *t*_0_ with a dispersion τ in time. The transient response $R_{T}$ is simply taken to be the first derivative of the sustained response $R_{S}$. Figure 2A illustrates the behavior of the temporal receptive fields when convolved with a frequency modulated chirp stimulus. When the chirp stimulus is convolved with the sustained (blue) and transient (red) temporal receptive fields, the relative amplitude of the resulting signals varies according to the frequency. The sustained response has large amplitude at low frequencies but then attenuates as the frequency increases. The transient response, on the other hand, shows weaker amplitude at low frequencies, but the amplitude becomes stronger as the frequency increases, and then eventually tapers off. Thus, information about the temporal frequency tuning can be recovered from the amplitude modulation of the modeled responses. Theoretically, the spatiotemporal pRF model requires, at minimum, two flicker frequencies in order to differentiate the amplitudes of the stimulus responses. For the sake of efficiency, we chose two frequencies based on pervious work in the macaque LGN [20]. We embedded this frequency-encoded amplitude modulation into a sweeping bar stimulus that simultaneously encoded visuotopic location (Figure 2B). The responses of the temporal RFs to the bar flickering at 10 Hz and 20 Hz differ in their amplitude modulations where the sustained RF response is larger for 10 Hz compared to 20 Hz while the transient RF response is larger for 20 Hz compared to 10 Hz. Since there can only be one measured response from each voxel over time, it is necessary to combine the output of the sustained and transient temporal receptive fields. To accomplish this, we added a weight parameter for mixing the sustained and transient responses to produce a single weighted sum approximating the BOLD response. Owing to the relatively coarse sampling resolution of fMRI and the small size of the LGN and its laminar subdivisions, it is exceedingly unlikely that any given voxel would sample from a purely M or P population of neurons. With this term and the spatial and temporal model components, the full spatiotemporal pRF model is given as

$$R=\mu +\beta \times \int^{\;}S\left(x,y,t\right)*G\left(x,y\right)*\left[\omega *R_{S}\left(t\right)+\left(1-\omega \right)*R_{T}\left(t\right)\right]\;dx\;dy\;dt$$

where the modeled BOLD response R is taken to be the integral of the stimulus S, the Gaussian spatial receptive field G, the sustained and transient temporal receptive fields $R_{S}$ and $R_{T}$, the mixing parameter ω, the scaling parameter β, and the baseline parameter μ. Thus, we have three parameters (x,y,σ) describing the spatial tuning and one parameter (ω) describing the temporal tuning of each voxel. A similar spatiotemporal RF model has been used to describe direction selectivity in simple cells in macaque V1 [21] and temporal response properties in human cortex [22,23].

In addition to these model parameters, we also included other model parameters for capturing the delay of the HRF and dispersion of the temporal RF across the LGN within a single subject. We estimated the double-gamma HRF delay [24] parameter δ and the temporal RF dispersion parameter τ simultaneously among all voxels surviving a statistical threshold (*r*^2^ > 0.1) in a yoked fashion, so that while the 6 spatiotemporal pRF model parameters (x,y,σ,ω,β,μ) were estimated independently for each voxel in a given subject’s LGN, the model parameters δ and τ were estimated iteratively and in tandem across the entire LGN for each subject. Thus, all voxels shared the same HRF delay and temporal RF dispersion at each iteration of the gradient-descent procedure. This iterative procedure for yoking some parameters but independently estimating others is a crucial component of this analysis, as the dispersion parameter τ is degenerate with the transient-sustained mixing parameter ω. Yoking τ across all voxels allowed us to independently estimate the weight parameter ω across voxels. We then took the distribution of the weight parameter to be a description of the functional organization of the M and P responses across the extent of the LGN. We place no physiological meaning on the temporal RF dispersion parameter. The spatial response was convolved with the $R_{S}$ and $R_{T}$ temporal receptive fields, which in turn were rectified and combined according to the mixing parameter ω and convolved with the HRF (Figure 2C) to approximate the BOLD signal.

The goodness of fit between the model prediction and the measured BOLD signal was assessed via the residual sum of squared error (RSS). The time-series of each voxel was fitted in a two-phase procedure. The first phase consisted of a sparse and coarse global grid-search that adaptively constrained the search boundaries. The effective stimulus was down-sampled to a resolution of 5% of the original, using a two-dimensional bilinear interpolation. An adaptive brute-force search strategy was used to sparsely sample the model parameter space and the best fit of this was used as the seed point for a fine-tuned gradient descent error-minimization using the non-resampled stimulus. The gradient-descent procedure used was a downhill simplex algorithm [25] to fine-tune the parameter estimates. The spatial parameters were searched in the space of ±10° (full field) from fixation. The pRF model yields an explained variance for each voxel, which serves as the statistical threshold $\left(r^{2}>0.1\right)$. All pRF modeling was done using popeye, open-source software for estimating population receptive field models [26].

To assess the reliability of our pRF model estimates, we used a bootstrapping procedure where we repeatedly fit the pRF model to the voxel-wise mean time-series computed from a variety of functional run sample sizes. For every voxel in each subject’s LGN, we randomly sampled without replacement a number of runs (ranging from 2–29), estimated the pRF model, and computed an error score by computing the difference between each bootstrapped pRF model fit against the full 30-run pRF model estimate. We repeated this 200 times for each voxel and sample size. The error between the resampled pRF estimate and the target pRF estimate was compared across sample sizes and subjects.

### 2.6. Model-Free Functional Segmentation of the LGN

The spatiotemporal pRF model parameter ω provides a measure of the relative contribution of transient and sustained temporal responses. The parameter estimate varies between 0 and 1, with 0 being a purely transient response and 1 being a purely sustained response. The distribution of the ω parameter estimates across the LGN and then represents a functional gradient rather than a functional segmentation per se. In order to determine if the distribution of ω estimates across the LGN emanates from the M and P response properties, we designed full-field stimuli that varied stimulus properties over time so as to maximally differentiate the presumptive signal of the M and P layers. Voxels containing a preponderance of M or P neurons should show a unique pattern of BOLD activation depending on the flicker frequency, the spatial frequency, and departures from red-green isoluminant reference point. We used two model-free approaches to classify voxels on the basis of the BOLD response to the full-field stimuli. We included all voxels from our hand-drawn anatomical LGN ROIs in the clustering analysis. The BOLD time-series of the surviving voxels from the two full-field experiments were concatenated in time and a dissimilarity matrix was computed by taking the pairwise Euclidean distance of the correlation matrix. This dissimilarity matrix was then clustered using an agglomerative hierarchical clustering algorithm [27], specifying a cluster size of three. We reasoned that each pair of LGN should cluster into two compartments representing the M and P responses, and a third compartment representing portions of the LGN not simulated by our visual stimulus projection system. In addition, we used a logistic regression to determine which pRF model parameter estimates best accounted for the cluster labels.

Uncorrected *p*-values are reported for all voxel parameter regressions. FMRI Voxels are typically not independent; however, the BOLD point spread function (PSF) in the LGN is much smaller than in the cortex—the best estimate is 1.6 mm [4,28]. In the cortex, the PSF is largely driven by the horizontal connections rather than BOLD spreading [29,30]. Therefore, the original voxels as acquired in our samples are essentially independent, but a correlation is introduced during the resampling by a factor of 2 in each dimension, and the alpha value for comparisons should correspondingly be increased by a factor of 8.

## 3. Results

### 3.1. Spatiotemporal pRF Model Estimates

Table 1 shows the mean volumes for the LGN ROIs among our subjects, divided by hemisphere. The use of PD images to guide the drawing of the LGN ROIs was critical, as the T1-weighted anatomical images did not offer sufficient contrast to identify the boundaries of the LGN. We have previously reported the detailed hand-drawn LGN ROIs for these three subjects in a separate study [1] and a number of other subjects across two other studies [5,31]. Table 1 also shows the activated volumes among our subjects and hemispheres, discounting voxels that did not survive the activation threshold $\left(r^{2}>0.1\right)$ computed by regressing the measured BOLD response against the modeled pRF response.

**Figure 3:**
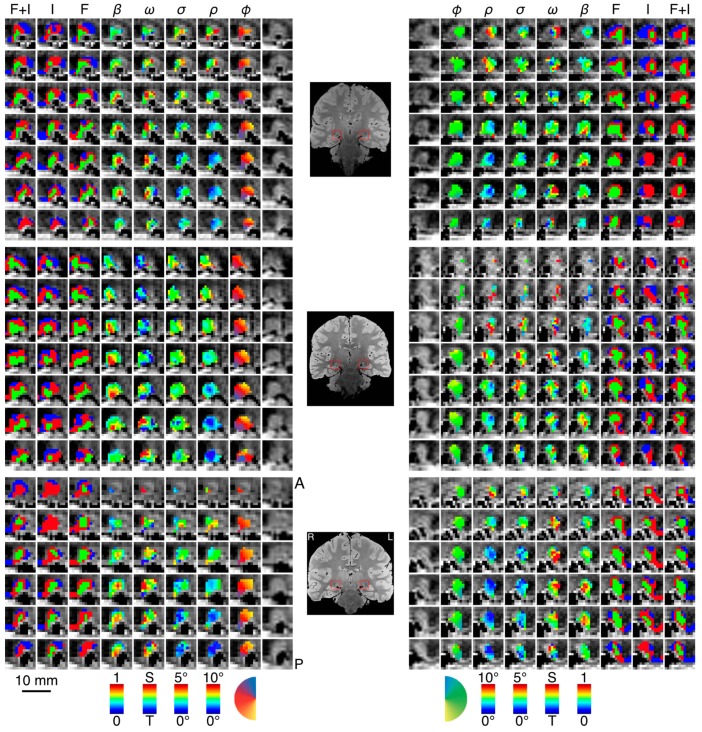
Parameter maps across three subjects. In each panel—top, middle, bottom—we show the left and right LGN for a single subject with the spatiotemporal pRF model estimates overlaid atop the proton-density image. We show a series of contiguous coronal slices moving from anterior to posterior along the rows from top to bottom. The innermost column shows the unlabeled anatomy, followed by the polar angle, the eccentricity, the receptive field size, the transient-sustained mixture, and amplitude. The three outermost columns show the cluster labels derived from data while subjects viewed the full-field flicker stimulus, the full-field isoluminant stimulus, and the full-field flicker and isoluminant concatenated in time. Color maps for each of the parameters are presented at the bottom of the figure below each column. A single coronal slice from each subject’s proton-density image is shown between the left and right hemisphere panels. The left and right LGN are highlighted with a red box. Labels at the top of the figure indicate the parameter (*φ*, polar angle; *ρ*, eccentricity; *σ*, receptive field size; *ω*, transient-sustained weight; *β*, amplitude; F, flicker cluster labels; I, isoluminance cluster labels; F + I, concatenated flicker and isoluminance cluster labels).

**Figure 4:**
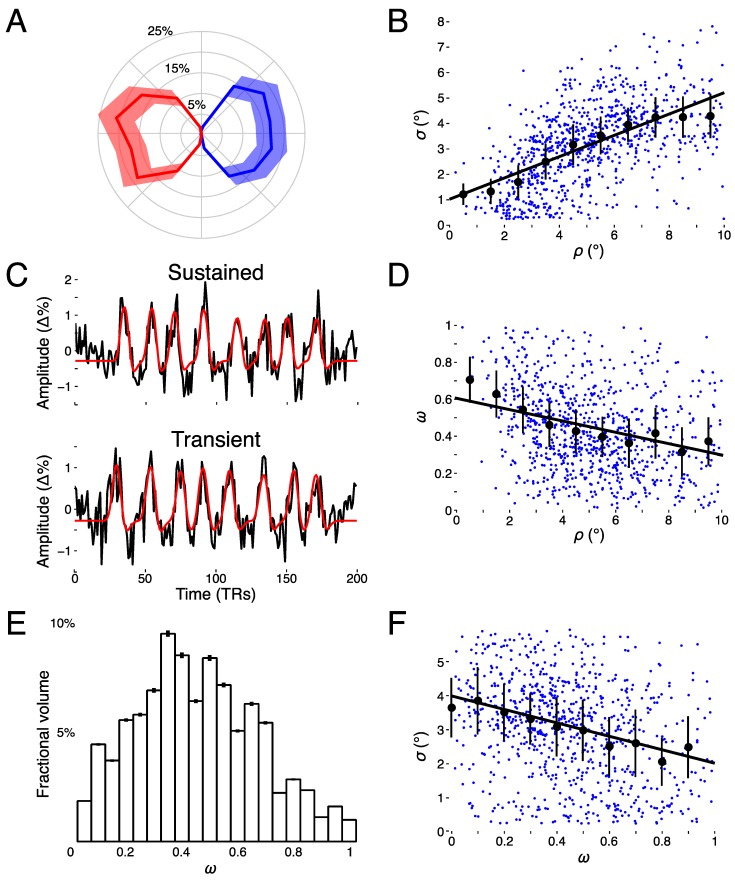
Model estimates. (**A**) Polar angle as a function of fractional volume for left (blue) and right (red) LGN. (**B**) Receptive field size (*σ*) as a function of eccentricity (*ρ*). (**C**) Example voxel data and model fits for a sustained, parvocellular-like (top panel) voxel and a transient, magnocellular-like (bottom panel) voxel time-series [percent signal change (Δ%)]. (**D**) Transient-sustained weight (*ω*) as a function of eccentricity (*ρ*). (**E**) Distribution of transient-sustained weights as function of fractional volume. (**F**) Receptive field size (*σ*) as a function of transient-sustained weight (*ω*).

**Figure 5:**
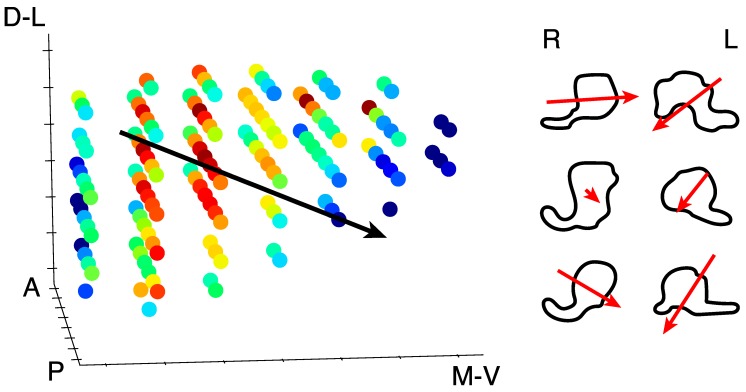
Weight parameter gradient. Left panel: All of the voxels in one LGN are shown to highlight the gradient of the transient-sustained weight parameter. Red voxels exhibit sustained responses whereas blue voxels exhibit a transient response. The black line indicates the direction of the gradient from sustained to transient through the whole LGN, from dorsolateral (D-L) to ventromedial (V-M). Anterior (A) and posterior (P) directions are also labeled. Right panel: The right (R) and left (L) LGN from each of the three subjects are shown outlined in the coronal plane. The red arrows indicate the direction and magnitude of the gradient from sustained to transient.

We found clear bilateral retinotopic maps in the LGN of each of our three subjects. Figure 3 shows the pRF model parameter maps for three subjects (top, middle, and bottom panels). Each panel is subdivided into columns depicting the pRF model parameters estimates and rows representing consecutive coronal slices through the LGN. We converted the location parameters from Cartesian to polar coordinates. Thus, the columns show model estimates for the polar angle, eccentricity, receptive field size, amplitude, and weight parameters. In addition, we also show the unlabeled anatomy. Only voxels exceeding the activation threshold are shown in the functional maps. The retinotopic organization of the LGN across the three subjects is in close agreement with our previous findings [1]. Each LGN contains a representation of the contralateral hemifield, with the upper vertical meridian represented along the inferior-lateral boundary of the LGN, the lower vertical meridian represented along the superior-medial boundary of the LGN, and the representation of the contralateral horizontal meridian oriented at approximately 45° subdividing the upper and lower visual field representations in each LGN. Figure 4A shows the polar angle representation of the LGN pooled among our three subjects. Here, we plot the fractional volume as a function of polar angle separately for the left (blue) and right (red) hemispheres separately, using sixteen 22.5° radial segments. The solid lines represent the mean fractional volume and the shaded region represents the standard error of the mean (SEM). All LGN activated bilaterally and showed strong contralateral representation of the visual space. In addition, the LGN showed a representational bias such that the horizontal meridians were over-represented and the vertical meridians were under-represented. This representational bias of the retinotopic organization has been shown previously in the LGN and in a number of other visual subcortical nuclei [1,2,3,4,32]. The eccentricity maps among our subjects showed a similar consistency among subjects and hemispheres and with our previous reported spatial pRF model estimates in the human LGN. Namely, we found that foveal representations near the posterior pole of the LGN and peripheral representations near the anterior pole of the LGN, with a smooth gradation of eccentricity representations along the anterior-posterior anatomical gradient. Likewise, the pRF size maps showed a similar anterior-posterior gradient with small receptive fields near the fovea and large receptive fields in the periphery. Figure 4B shows the relationship between eccentricity and receptive field size. Here, we plot the spatiotemporal model parameter σ as a function of eccentricity, showing the mean (black dots) and SEM (black bars) binned into ten 1° concentric segments. In addition, we show the raw data for all voxels (blue dots) and the linear fit (black line) through all points (*r*^2^ = 0.29, *p* < 0.0001). We also found orderly maps of the amplitude and weight parameters. The amplitude maps represent the multiplicative scaling factor used to approximate the unit scale of the measured BOLD response. We found that the amplitude maps were consistent across subjects and hemispheres such that the voxels with the largest amplitudes were clustered in the hilum region, the central medioventral portion of each LGN, and that the amplitude decreased towards the dorsolateral aspect of each LGN (Figure 3). The weight maps also showed consistency across subjects and hemispheres. Specifically, we found higher weights along the lateral aspect of the LGN and lower weights along the medial aspect of the LGN (recall that a weight of 0 is a purely transient temporal RF response while a weight of 1 is a purely sustained temporal RF response). Figure 4C shows the time-course of two voxels showing the measured BOLD response and the modeled pRF response. The top panel of Figure 4C shows the measured BOLD response (black) and the modeled pRF response (red) for voxels with sustained-rich (ω=0.83, top) and transient-rich (ω=0.17, bottom) mixtures. The first four peaks represent the measured (black) and modeled (red) responses to the first set of bar sweeps flickering at 10 Hz. The second set of four peaks represent the measured and modeled responses to the second set of bar sweeps flickering at 20 Hz. The parameter of the spatiotemporal pRF model captures the flicker-mediated amplitude modulation such that voxels with a large ω show large amplitude attenuation for the 20 Hz versus the 10 Hz visual flicker stimulation while voxels with a small ω do not show this attenuation across flicker conditions. Figure 4E illustrates the distribution of weights among our three subjects. We also show the relationship between eccentricity and weight (4D) and weight and receptive field size (4F). We found a negative relationship between the transient-sustained weight parameter and eccentricity such that voxels near the fovea tend to have sustained weight estimates while voxels in the periphery tend to have transient weight estimates (*r*^2^ = 0.07, *p* < 0.0001). We also found a negative relationship between the transient-sustained weight parameter and receptive field size such that voxels with small receptive fields tend to have sustained weight estimates while voxels with large receptive fields tend to have transient weight estimates (*r*^2^ = 0.04, *p* < 0.0001). Taken together, these results are congruent with previous work showing that P neurons have small receptive fields located near the fovea and exhibit sustained discharge patterns while M neurons have been shown to have large peripheral receptive fields with transient discharge patterns. However, despite the correlation between the weight and eccentricity parameters, the parameters show distinct organizations. In Figure 5, we show the gradient of the weight parameter from sustained to transient. This gradient is shown for one example LGN in the left panel in Figure 5. To compare this gradient across hemispheres and subjects, we also computed the direction of the sustained to transient weight gradient for each of the 6 LGN tested, as shown in the right panel in Figure 5. Each gradient vector is shown in the coronal plane superimposed on the outline of the central coronal slice of each LGN. To determine the consistency of the gradient across the 6 LGN, we projected each vector into the coronal plane and analyzed the angle from the horizontal using circular statistics [33]. The mean angle was 36.4° (95% confidence interval 16.7–56.0°) in the dorsolateral to ventromedial direction, as would be expected from the P to M organization and was significantly different from a random distribution (Rayleigh test, *p* = 0.0014).

**Figure 6:**
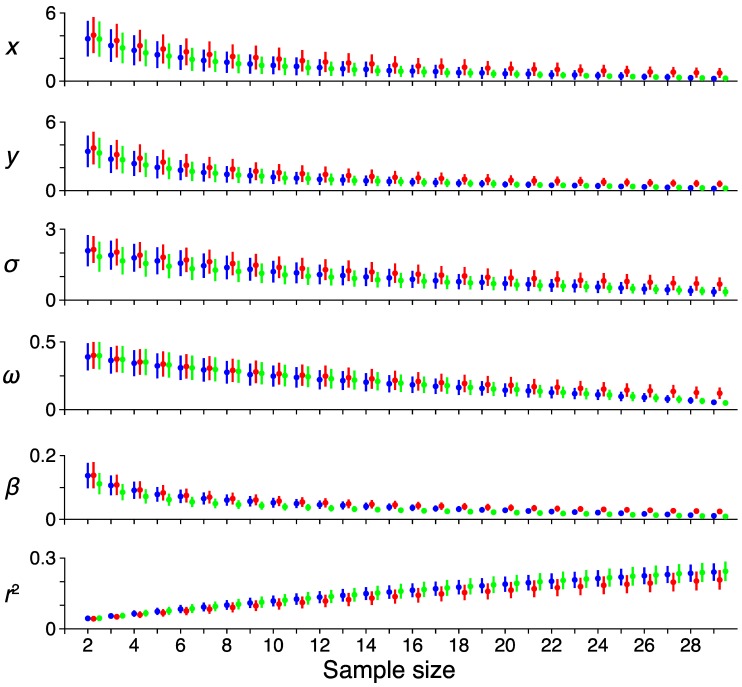
Reliability. The effect of increasing sample size on the error rates among the different pRF model parameters. Each color represents a single subject. The average error among all voxels for a sample size and the reference pRF estimate computed from all runs are shown as a dot, with error-bars showing the 95% confidence intervals. Each row shows a different pRF parameter (*x*, horizontal location; *y*, vertical location; *σ*, receptive field size; *ω*, transient-sustained weight; *β*, response amplitude; *r*^2^, variance explained).

To test the reliability of the pRF model parameter estimates, we performed a bootstrapping procedure for tracking the variability of the model fits as the number of samples increased. Figure 6 shows the results of this analysis, where the mean deviation across voxels (dot) and variability (bars, 95% confidence intervals) is shown across sample sizes for the pRF estimates and the variance explained. Each color represents a single subject. As the number of samples increases, the error of each voxel decreases, while the explained variance increases. The weight parameter shows a shallower decline in error with increasing sample size compared to the other parameters, suggesting that this model term is most susceptible to noise.

### 3.2. Model-Free Data-Driven Segmentation of the LGN

The weight parameter of the spatiotemporal pRF model provides a continuous measure of the relative contribution of the M and P neural subpopulations towards the aggregate BOLD signal. Since it is exceedingly unlikely that any given voxel would sample from a purely M or P neural population, the weight parameter represents a functional gradient rather than a functional clustering. Voxels may have a weight estimate indicative of a P population with a sustained discharge pattern or an M population with a transient discharge pattern. However, since we have no clear a priori threshold for binarizing our weight map into an M/P map, we sought to independently segment the LGN on the basis of the functional activity in response to a number of relevant stimulus features. We presented subjects with full-field stimuli in order to remove any spatial component from the BOLD signal and varied the stimulus properties to maximally differentiate the presumptive M and P responses underlying the BOLD signal.

We performed a logistic regression to determine which of the pRF model parameters best explained the cluster labels derived from the full-field datasets (Table 2). A regression of the pRF estimates against the cluster labels derived from the full-field flickering stimulus [*F*(6,581) = 13.67, *p* < 0.0001, *r*^2^ = 0.12] showed that the eccentricity (*β* = −0.04, *p* < 0.01) and amplitude (*β* = −0.07, *p* < 0.0001) best explained the variation in the model-free labeling of the LGN. Regression of the pRF estimates against the cluster labels derived from the full-field isoluminance stimulus [*F*(6,396) = 42.33, *p* < 0.0001, *r*^2^ = 0.40] revealed that eccentricity (*β* = −0.09, *p* < 0.01), receptive field size (*β* = 0.12, *p* < 0.01), transient-sustained weight mixture (*β* = −0.10, *p* < 0.01), and amplitude (*β* = −0.31, *p* < 0.0001) all significantly covaried with the cluster labeling in the LGN. Finally, we regressed the pRF estimates against the full-field flicker and isoluminance stimuli concatenated in time [*F*(6,470) = 32.86, *p* < 0.0001, *r*^2^ = 0.30]. We found that the amplitude (*β* = −.17, *p* < 0.0001) and baseline (*β* = −0.05, *p* < 0.01) both significantly covaried with the cluster labeling in the LGN. Interestingly, there was no significant correlation between our weight and amplitude estimates [*r*(915) = −0.02, *p* = 0.35], indicating that these parameters are unrelated.

## 4. Discussion

The pRF model has been used to explore and describe the visual responses measured in a number of subcortical [1] and cortical areas [16,34,35,36] using functional imaging techniques. Since its inception, it has proved to be a robust and expressive model for capturing and formulating the tuning properties throughout the visual pathway. The Gaussian pRF model is supplanting the phase-encoding retinotopic mapping [37,38,39] approach. Phase-encoding analyses were important in the early years of fMRI due to their efficiency in the light of the lower SNR of imaging hardware and also the lack of computational power for analysis. By analyzing the steady state of stimulus-induced hemodynamic oscillations, the Fourier analyses are not sensitive to the exact form of the hemodynamic response function. However, the spatial parameters, e.g., the polar angle and eccentricity activation coordinates in the visual field, must be measured in separate scanning runs, and the phase-encoding may introduce biases, especially in the presence of noise [40]. PRF modeling in contrast can be applied to arbitrary stimuli and can encode multiple features in parallel. We sought to extend the pRF model into the temporal domain in an effort to explore the response properties of the human LGN, and to segment it on the basis of its function.

We developed a spatiotemporal pRF model that includes both spatial and temporal receptive fields to capture differences in the response properties of neurons in the M and P layers of the LGN. While difficult to measure because of its small size and deep location in the brain, the unique functional and structural organization of the LGN offers a rich experimental arena in which to develop and test models of information flow and dysfunction throughout the visual system. Among their distinguishing characteristics, the M and P layers of the LGN are dissociable based on their response profiles to various stimuli. In particular, we sought to exploit the differences in the temporal frequency tuning and discharge patterns of M and P neurons. We extended the spatial pRF model to include a pair of temporal receptive fields, where the ideal P response was modeled as a Gaussian in time and the M response was modeled its first derivative. These impulse response functions were deliberately chosen to be simple and were not tuned to optimize the discrimination of the M and P layers of the LGN. The aggregate BOLD response was taken to be the weighted sum of the transient and sustained responses, in combination with the spatial and hemodynamic components. The functional form of the temporal RF helps to link the neural response properties of temporal frequency tuning and the discharge pattern. A transient discharge pattern—the rapid cycling of neural activity—acts as an edge detector in time and so confers the property of high temporal frequency tuning. A sustained discharge pattern—tonic activation during the presentation of a stimulus—acts as a low-pass temporal filter and confers the property of low temporal frequency tuning. When a stimulus with varied flicker frequencies is passed through the temporal RFs, the resulting response will demonstrate a frequency-driven amplitude modulation over time. Thus, the spatiotemporal pRF model estimation affords information about how a given voxel is tuned in space and time.

Although temporal frequencies could be analyzed using a traditional GLM, the advantage of the pRF model is that we can apply the model to analyze the response to any stimulus, rather than having to craft a particular stimulus for each feature we would like to analyze. In addition, we could readily add other response features to the model, such as color or eye of origin. Each feature could be measured independently using a GLM, but if the stimulus features are separated in the stimulus, confounding factors such as attention can interfere. Additional theoretical questions could be addressed by the pRF but not GLM models such as questions of spatial pooling and differential center-surround temporal modulations.

The retinotopic organization of our LGN maps is consistent with our previous work. All LGN showed strong contralateral representation of the visual field, with the lower vertical meridian representation along the superior-medial LGN, the upper vertical meridian representation along the inferior-lateral LGN, and the contralateral horizontal meridian bisecting the two about approximately 45°. The eccentricity and receptive field size maps showed a strong positive relationship such that voxels at the posterior pole of the LGN had small, foveal receptive fields and voxels at the anterior pole of the LGN had large, peripheral receptive fields. These findings demonstrate that the spatial component of the spatiotemporal model can be faithfully estimated with a stimulus that encodes multiple features.

In addition to the spatial tuning, we were also able to recover the topographic organization of the temporal tuning across the LGN. Among our three subjects, we found a smooth gradation of the weight parameter estimates along the lateral-superior to medial-inferior axis of the LGN. Specifically, we found that voxels along the lateral-superior portion of the LGN contained voxels with sustained-rich weight estimates and voxels along the medial-inferior portion of the LGN contained voxels with transient-rich weight estimates. The configuration and orientation of these weight parameter maps is generally congruent with the known structural and functional organization of the LGN.

Previous functional imaging experiments aimed at dissociating the M and P layers of the human LGN found a similar organization of the regression coefficients computed from a GLM using a battery of M-preferred and P-preferred stimuli [8,9,10] including spatial frequency, temporal frequency, color, and contrast. The experiments relied on the centers of mass of the beta weights associated with M-preferred and P-preferred stimuli to determine the anatomical axis of the functional topography. We find these approaches problematic for several reasons. First, LGN regions of interest (ROIs) were determined on the basis of the functional activation. Since the LGN is flanked by several visually responsive structures including the triangular area, the pulvinar and blood vessels, functional definition of the LGN will invariably result in the inclusion neighboring voxels not belonging to the LGN. In the present study, each subject participated in an anatomical scanning session where 40 proton-density weighted images were collected, registered, and averaged. These data provided a clear anatomical image for determining the location and extent of the LGN without relying on functional measures. Second, these previous studies employed a GLM analysis that is unable to distinguish between hemodynamics and function. Our amplitude maps, which estimate the hemodynamic gain and not differential functional properties, have a similar topography to GLM beta coefficient maps reported in previous studies [8,9,10,41]. It may be that these previously reported M/P maps are extracting information about the gain of the BOLD response due to the hilum since their regression coefficient maps and our amplitude maps show greatest amplitudes there. Any GLM approach that involves the subtraction or contrast between the beta-weight amplitudes evoked by two different experimental conditions (e.g. [8]) is likely to find maximal differences in the hilum for any stimuli, as the amplitude changes are intrinsically the largest there. This hemodynamic confound needs to be accounted for to be able to make any functional inferences. One way to do this using a GLM approach would be to compute a modulation index, e.g., (*β*_1_−*β*_2_)/(*β*_1_+*β*_2_) instead of analyzing the absolute difference *β*_1_−*β*_2_. The pRF modeling approach we propose explicitly includes both the transient-sustained components of the BOLD signal as well as the amplitude of the BOLD signal for each voxel. That we found no correlation between the weight and amplitude parameters suggests that the weight parameter is sensitive to the temporal response properties across the extent of the LGN, independent of the changes in amplitude.

Other recent work has focused on characterizing the effects of achromatic flicker frequency on the BOLD response in human primary visual cortex (V1) and the LGN [42]. In this study, the authors present data indicating that the BOLD response in the LGN is relatively insensitive to differences in achromatic flicker frequencies ranging from 6–46 Hz. In our present study, we were able to distinguish differences in the BOLD response amplitude to two achromatic flicker frequencies (10 Hz and 20 Hz). One possible reason for the differences between our findings is that these researchers pooled the BOLD response across the entire functionally defined LGN ROIs. Since it is known that there are differences in the temporal response properties of M and P neurons distributed across the layers of the LGN, the lack of flicker frequency encoded BOLD amplitude modulation may be lost when the signal is averaged across the M and P layers. In our study, we estimate this frequency encoded amplitude modulation for each voxel individually and include a transient-sustained mixture parameter to account for single voxels containing both M and P neurons.

To determine how much of the variation in the activity of the LGN the spatiotemporal pRF model captures, we performed two additional experiments. Here, we removed any spatial component from the stimulus and presented subjects with full-field checkerboard stimuli whose flicker frequency, spatial frequency, and red-green isoluminance were varied so as to maximally differentiate the underlying M and P response in the LGN. We then used model-free clustering and decomposition techniques in order to determine if we could replicate the topographies of the pRF model parameter estimate maps obtained in Experiment 1. The results of our hierarchical clustering revealed a functional organization of the cluster labeling that roughly agreed with our amplitude estimate maps. We performed several regressions to determine the extent to which the parameter estimates of the spatiotemporal pRF model explain the variation in the cluster labels derived from full-field flickering and isoluminance data, and the concatenation of these two datasets. While we found small variations in the cluster label organization across the different cluster label experiments, we did find that there was a consistent significant relationship between the cluster labels and the amplitude pRF model estimate. We interpret the amplitude to represent the hilum region of enriched vasculature in the center of the medioventral surface of the LGN [11]. The amplitude of the BOLD signal may drive the boundaries of the cluster labels rather than any intrinsic differences in the functional properties of population of M and P neurons, as the results of the regression analyses suggest. Explicitly modeling the amplitude with the spatiotemporal pRF model provides an analytical strategy for sequestering the variation due to BOLD amplitude variation. Functional brain imaging experiments measuring the functional response properties of the LGN should consider variation in BOLD amplitude across the extent of the LGN, otherwise vascular effects can be mistaken for functional properties.

Part of the motivation for developing methods to parse the layers of the LGN was to provide a framework to test the magnocellular theory of dyslexia. However, the substantial amount of data collected for these experiments may limit the viability of using such an approach in a clinical setting. To address this issue, we conducted a bootstrapping procedure where we estimated the stability of the spatial and temporal pRF model parameters as sample sizes increases. We found that the stability of the spatial parameters quickly saturated after approximately 10 resamples, and the temporal parameter reached 90% stability after approximately 20 scanning runs. In other words, 75–100 min of functional scan time is required to detect the transient-sustained functional gradient of the human LGN using a statistical threshold of *r*^2^ > 0.1. This could potentially place irreconcilable time constraints on clinicians scanning patients, although modifications could be made to the stimulus regime to sacrifice spatial precision for temporal sensitivity. Furthermore, only approximately one-third of the voxels in the anatomically defined LGN survived the statistical threshold. This represented about 25% reduction compared to a previous study using pRF modeling in the LGN [1]. The stimuli we used covered only a fraction of the visual field, so we would expect only a portion of the LGN voxels to be active. Also, the activated volume is determined by the threshold, which is a function of many factors, including scanner noise, head-movement, eye-movements, subject vigilance, and stimulus duty cycle. One simple strategy one might adopt to increase detectability is to increase the duty cycle of the stimulus. In the current experiment, the duty cycle of the bar was set to 0.25, meaning that any given pixel in the viewable display was “on” for 25% of the scanning time. In our previous study, the duty cycle of the bar stimulus was 0.5, meaning that every pixel half the scanning time. While this approach is the most efficient, using such a large bar stimulus may reduce the precision of the pRF size estimates.

## 5. Conclusions

We have developed a spatiotemporal pRF model to account for the responses of the human LGN. We found that the response amplitude is dominated by the rich vascular hilum region, which drives unsupervised clustering attempts. Once this amplitude was parameterized, we were able to measure a gradient of temporal sustained to transient responses that can be attributed to the parvocellular and magnocellular subdivisions of the LGN. The temporal pRF model is flexible and simultaneously accounts for multiple parameters for a given stimulus, and additional parameters accounting for other stimulus parameters or neural features can be added.

## Figures and Tables

**Table 1:** The mean volume (mm^3^) of the left and right LGN ± subject-wise SEM. Also shown are the mean activated volumes after applying the statistical threshold.

	Left Hemisphere	Right Hemisphere
LGN	255 ± 14	251 ± 22
LGN, *r*^2^ > 0.10	69 ± 2	65 ± 6

**Table 2:** Regression results. We regressed each of the spatiotemporal pRF model estimates against the intrinsic functional clusters derived from data measured while subjects viewed a full-field flicker and isoluminant stimuli. Beta coefficients are standardized.

	Flicker			Isoluminance			Flicker + Isoluminance		
pRF Parameter	*β*	SE	*p*-Value	*β*	SE	*p*-Value	*β*	SE	*p*-Value
*φ* polar angle	0.02	0.012	0.086	0.02	0.02	0.44	0.04	0.01	0.016
*ρ* eccentricity	−0.04	0.013	<0.01	−0.09	0.02	<0.01	−0.03	0.02	0.079
*σ* receptive field size	−0.03	0.017	0.035	0.12	0.03	<0.01	−0.05	0.02	0.012
*ω* weight	−0.004	0.013	0.78	−0.10	0.03	<0.01	−0.03	0.02	0.061
*β* amplitude	−0.07	0.014	<0.0001	−0.31	0.02	<0.0001	−0.17	0.01	<0.0001
*μ* baseline	0.03	0.012	0.015	0.05	0.02	0.019	0.05	0.01	<0.001
